# Celiac disease T-cell epitopes from gamma-gliadins: immunoreactivity depends on the genome of origin, transcript frequency, and flanking protein variation

**DOI:** 10.1186/1471-2164-13-277

**Published:** 2012-06-22

**Authors:** Elma MJ Salentijn, D Cristina Mitea, Svetlana V Goryunova, Ingrid M van der Meer, Ismael Padioleau, Luud JWJ Gilissen, Frits Koning, Marinus JM Smulders

**Affiliations:** 1 Plant Research International, Wageningen UR, P.O. Box 16, NL-6700 AA, Wageningen, The Netherlands; 2 Wageningen UR Plant Breeding, Wageningen, The Netherlands; 3 Leiden University Medical Centre, Leiden, The Netherlands; 4 Vavilov Institute of General Genetics, Russian Academy of Sciences, Moscow, 119991, Russia

**Keywords:** Wheat, Gluten, γ-gliadins, Celiac disease, T-cell epitopes

## Abstract

**Background:**

Celiac disease (CD) is caused by an uncontrolled immune response to gluten, a heterogeneous mixture of wheat storage proteins. The CD-toxicity of these proteins and their derived peptides is depending on the presence of specific T-cell epitopes (9-mer peptides; CD epitopes) that mediate the stimulation of HLA-DQ2/8 restricted T-cells. Next to the thoroughly characterized major T-cell epitopes derived from the α-gliadin fraction of gluten, γ-gliadin peptides are also known to stimulate T-cells of celiac disease patients. To pinpoint CD-toxic γ-gliadins in hexaploid bread wheat, we examined the variation of T-cell epitopes involved in CD in γ-gliadin transcripts of developing bread wheat grains.

**Results:**

A detailed analysis of the genetic variation present in γ-gliadin transcripts of bread wheat (*T. aestivum*, allo-hexaploid, carrying the A, B and D genome), together with genomic γ-gliadin sequences from ancestrally related diploid wheat species, enabled the assignment of sequence variants to one of the three genomic γ-gliadin loci, *Gli-A1*, *Gli-B1* or *Gli-D1*. Almost half of the γ-gliadin transcripts of bread wheat (49%) was assigned to locus *Gli-D1*. Transcripts from each locus differed in CD epitope content and composition. The *Gli-D1* transcripts contained the highest frequency of canonical CD epitope cores (on average 10.1 per transcript) followed by the *Gli-A1* transcripts (8.6) and the *Gli-B1* transcripts (5.4). The natural variants of the major CD epitope from γ-gliadins, DQ2-γ-I, showed variation in their capacity to induce *in vitro* proliferation of a DQ2-γ-I specific and HLA-DQ2 restricted T-cell clone.

**Conclusions:**

Evaluating the CD epitopes derived from γ-gliadins in their natural context of flanking protein variation, genome specificity and transcript frequency is a significant step towards accurate quantification of the CD toxicity of bread wheat. This approach can be used to predict relative levels of CD toxicity of individual wheat cultivars directly from their transcripts (cDNAs).

## Background

Gluten proteins present in wheat can cause celiac disease (CD) [1]. Wheat gluten consists of a complex mixture of α/β-, γ- and ω-gliadins, and high and low molecular weight (HMW; LMW) glutenins, which are all encoded by medium to large multigene families. In contrast to other dietary proteins, gluten proteins are minimally digested by gastrointestinal proteases, resulting in relatively large peptides that accumulate in the small intestine [2]. In CD patients, during passage through the mucosal tract these gluten fragments can bind to the HLA-DQ2/8 receptors present on antigen presenting cells (APCs) and trigger T-cell responses. This T-cell response to HLA-DQ2/8 restricted gluten peptides occurs only in celiac patients and not in healthy controls. The HLA-DQ2/8 receptor favors peptides that contain one or more amino acids with a negative charge [3-10]. These are not present in gluten peptides but can be introduced due to the activity of human tissue transglutaminase (TG2) [11-14] an enzyme that converts glutamine (Q) residues into negatively charged glutamic acid (E) residues. Gliadin peptides, with their high proline and glutamine content, are perfect substrates for the transglutaminase reaction of TG2, which is critical for the creation of active T-cell epitopes involved in CD (CD epitopes). Since target sites for TG2 may be determined by sequences flanking the core motif, the amino acid composition of the surrounding of the epitope cores can influence the immunogenic capacity of a peptide regarding CD [8,13,14].

Several HLA-DQ2 and -DQ8 restricted T-cell epitopes have been identified in wheat gluten and in homologous proteins from barley (hordeins) and rye (secalins) ([7,8,15-26]. A 33-mer peptide, resistant to the action of proteases, derived from α-gliadins is regarded as one of the most CD-immunodominant gluten peptides [2,27]. However, there is clear evidence that γ-gliadins also contain T-cell stimulatory peptides [7,8,19,22,24,27-29]. In several specific cohorts a high frequency of CD patients was observed that mainly reacted to γ-gliadin peptides. Vader et al. [22] and Camarca et al. [24] found that half of the CD patients (respectively 10 of 20 children and 7 of 14 adults from Southern Europe) did not respond to peptides derived from α-gliadins, but instead reacted to peptides derived from γ-gliadins and LMW glutenins. A major CD epitope derived from γ-gliadins is the DQ2-γ-I epitope (PQQSFPQQQ[17,19]), recognized by one third of the patients [22,24]. Among pasta and bread wheat varieties differences have been observed in the capacity to stimulate *in vitro* DQ2-γ-I specific T-cell clones [30,31]. These facts justify an elaborated investigation of the γ-gliadins with special regard to the natural variation in CD-epitope content.

The γ-gliadins account for approximately 12% of the flour proteins of the hexaploid wheat cultivar Butte 86 [32]. They are encoded by clusters of tightly linked genes present at the *Gli-1* loci (*Gli-A1*, *Gli-B1* or *Gli-D1*) that are located on the short arms of the respective homoeologous group 1 chromosomes (1AS, 1BS and 1DS) of hexaploid bread wheat (*T. aestivum* L.) [33] and are tightly linked to the *Glu-3* (LMW glutenins) and *Gli-3* (ω-gliadins) loci [[34], and references therein]. The number of different γ-gliadin genes in the genome of bread wheat was estimated at 15–40 [35,36] and these can be clustered into four up to eleven groups [36-41]. This diversity is qualitatively reflected at the protein level [42], but not all genes are expressed evenly. Epitope content of the genes varies [39], just as it varied among α-gliadins [31,43-45] according to the genome of origin of the loci.

In the present study CD epitopes from γ-gliadins of bread wheat are considered in their natural context of deduced protein variation, TG2 deamidation sites, transcript frequency and genome specificity. For this purpose, we sequenced genomic γ-gliadin sequences from ancestrally related diploid *Triticum*/*Aegilops* species and analyzed these together with expressed γ-gliadin sequences from the NCBI database of hexaploid bread wheat (*Triticum aestivum* subspec. *aestivum*, with the A, B and D genome) to accomplish the assignment of γ-gliadin transcripts contigs to one of the three genomic γ-gliadin loci, *Gli-A1, Gli-B1* or *Gli-D1*, as was previously done for the α-gliadins [43,46]. Furthermore, natural occurring variants of a major γ-gliadin CD epitope, DQ2-γ-I, were analysed using T-cell assays.

## Methods

### Plant material

The taxonomy of *Triticum* and *Aegilops* is complex, and different taxonomic systems are being used. In this paper we have followed the classification according to Van Slageren [47]. Bread wheat (*T. aestivum* subsp. *aestivum*; 2n = 6x = 42; ABD genome) is an allohexaploid that was formed through hybridization and successive chromosome doubling of three diploid *Triticum/Aegilops* species. The diploid ancestors of the D genome and the A genome of bread wheat are respectively *Ae. tauschii* (D) and *T. urartu* (A^u^). *Ae. speltoides* var*. speltoides* (S) species or closely related species have been suggested as the ancestor of the B genome, but the exact diploid progenitor remains uncertain [48-50].

We included the following accessions in this study: *Ae. speltoides* var. *speltoides* (CGN 10682, according to the passport data originating from Israel, and CGN10684, originating from Turkey) is related to the B genome, *Ae. tauschii* (к-1368, Uzbekistan) as a D genome representative, and *T. monococcum* subspec. *monococcum* (CGN10542, A^b^ genome) as a representative of the A genome. The accessions were obtained from the Centre of Genetic Resources (CGN), The Netherlands, except K-1368, which is from N.I. Vavilov All-Russian Scientific Research Institute of Plant Industry (VIR), St. Petersburg, Russia.

At 21 days after anthesis, wheat kernels in the late milk ripening stage (250 mg) were grinded in liquid nitrogen and subsequently DNA was extracted with chloroform/isoamylalcohol as described by Van Herpen et al. [43].

### Amplification, cloning and sequencing of genomic γ-gliadin sequences

Primers to amplify γ-gliadin genes from genomic DNA using PCR were designed on the conserved sequences at the 5’ and 3’ end of the coding region of γ-gliadin gene sequences obtained from the EMBL database (forward primer, γ1F: 5’-atgaagaccttactcatcc-3’, and reverse γ11R: 5’-ggacaWagacRttgcacatg-3’). Primers were degenerated to cover as many different γ-gliadin sequences as possible. Amplification was performed in a 25 μl reaction volume, containing 0.2 μM reverse and 0.2 μM forward primer, dNTP mix (0.25 mM each), 1 x Pfu buffer (Stratagene) and a 1:4 (v/v) mixture of Pfu DNA polymerase (Stratagene) (2.5 U/μl) and Goldstar DNA polymerase (Eurogentec) (5 U/μl). Twenty ng of genomic DNA was used as a template for PCR. The PCR programs consisted of 5 min at 94°C followed by 24 cycles 94 °C for 1 min, 53°C for 1 min and 72°C for 2 min with a final extension at 72°C for 10 min. The PCR products were ligated into the pCRII-TOPO vector (Invitrogen) and subsequently transferred into *E. coli*-XL1-blue cells (Stratagene). Recombinants were identified using blue-white color selection. Positive colonies were picked and grown overnight at 37°C in freeze media (36 mM K_2_HPO_4_, 13.2 mM KH_2_PO_4_, 1.7 mM trisodium citrate, 0.4 mM MgSO_4_, 6.8 mM (NH_4_)_2_SO_4_, 4.4% v/v glycerol, 100 μg/ml ampiciline, 10 g/l tryptone, 5 g/l yeast extract and 5 g/l NaCl). The cloned insert was amplified directly from the culture in a PCR reaction using the M13 forward (5’-cgc cag ggt ttt ccc agt cac gac-3’) and reverse primer (5’-agc gga taa caa ttt cac aca gga -3’) in 20 μl reaction volume containing 2 μl of culture. The reaction mixture consisted of the same components as well as concentrations, and utilized the same PCR program as described before (annealing at 55°C). The amplified product was used in sequencing reactions using γ1F or γ6R(new) primers. Additional primers were designed on two internal, conserved regions of the γ-gliadin gene to generate additional sequences: one internal forward primer, γFi2: 5’-ccc(ac)tgcaagaat(at)t(ct)c-3’, and one internal reverse primer, γRi2: 5’-g(ag)a(at)attcttgca(gt)ggg-3’. The sequence data were manually checked using SeqMan (DNASTAR). In total, 69 unique γ-gliadin sequences have been deposited in the EMBL database (Table 1).

**Table 1:** Genomic γ-gliadin sequences

**Accession**	***Aegilops *spp.**	**genome**	**N**	**pseudogenes**	**Genbank accession**
CGN10684	*Ae. speltoides*	S	23	1	JQ269751-JQ269773
CGN10682	*Ae. speltoides*	S	15	2	JQ269774-JQ269788
A236	*Ae. tauschii*	D	15	1	JQ269789-JQ269803
CGN10542	*T. monococcum*	A^b^	16	6	JQ269804-JQ269819
			69	10 (6.9%)	

Number of obtained unique full open reading frame (full-ORF) sequences (N) and sequences with one or more stop codons (pseudogenes) from various diploid *Aegilops* / *Triticum* species. Accession numbers are given between brackets; Nomenclature according to Van Slageren [47]. *Ae. tauschii* = *Ae. squarrosa* k-1368, VIR, Russia.

### Neighbor-joining analysis of genomic γ-gliadin clones

The 69 genomic γ-gliadin sequences derived from the diploid wheat species *T. monococcum* subspec. *monococcum* (A^b^), *Ae. speltoides* var. *speltoides* (S) and *Ae*. *tauschii* (D), were included in alignment and distance analysis. All analyses were conducted in MEGA4 [51]. Complete sequences were trimmed to include a region spanning sequence domain I to V of the γ-gliadin protein. Due to the typical sequence repeats in the repetitive domains II and IV, the γ-gliadin sequences differed in length, ranging from 722 to 775 base pairs for *Ae. tauschii*, from 637 to 820 base pairs for *T. monococcum* subspec. *monococcum*, and from 725 to 968 base pairs for *Ae. speltoides* var. *speltoides*. Next, the trimmed sequences were analyzed using the Neighbor-Joining method [52]. The Neighbor-Joining method returned an optimal tree with the sum of branch length = 4.85106104. The sequence distances were computed using the Maximum Composite Likelihood method [53] in units of the number of base substitutions per site. All positions containing alignment gaps and missing data were eliminated only in pair wise sequence comparisons (Pairwise deletion option). There were a total of 968 positions in the final dataset. Codon positions included were 1st + 2nd + 3rd + non-coding. The number of base substitutions per site within species and between species was using respectively the Within-group function and Between-group function in MEGA4.

### Analysis and genomic assignment of γ-gliadin transcripts from bread wheat

A total of 939 γ-gliadin transcripts originating from *T. aestivum* subspec. *aestivum* were extracted from the NCBI database (on 13 November 2007). The γ-gliadin transcripts were assembled in 201 contigs of sequences with 98% to 99% sequence identity (SeqMan, Lasergene 8, DNAStar). The consensus nucleotide sequences of those contigs that were composed of at least four transcripts were further analyzed, resulting in 717 transcripts arranged in 47 contigs (ranging from 306 to 1038 nucleotides in length), coding for 26 different γ-gliadin protein isoforms, 10 of which are full length γ-gliadins (Additional file 1).

The γ-gliadins are encoded by clusters of tightly linked genes present at the *Gli-1* loci (*Gli-A1*, *Gli-B1* or *Gli-D1*) located on the short arms of the homoeologous group 1 chromosomes of bread wheat [33]. To determine whether actively expressed γ-gliadin sequences from bread wheat can be assigned to a genomic locus, based on sequence relationship to one of the diploid wheat species representing the ancestors of the bread wheat genomes, as was possible for α-gliadins [43], the sequences of the 26 expressed γ-gliadin isoforms detected in bread wheat were included in a Neighbor-Joining analysis together with different genomic γ-gliadins cloned from the diploid wheat species used in these studies as representatives for the ancestors of cultivated wheat. As the 26 different γ-gliadin isoforms were deduced from contigs of transcripts which are mostly partial sequences of expressed genes, not all of them covered the complete γ-gliadin gene. Therefore, a region of 333 to 384 base pairs in length, covered by all expressed isoforms, starting with domain III (NPC motif) and ending with domain V (MCN motif) was created and compared for sequence distances in a Neighbor-Joining analysis together with the same nucleotide region of genomic sequences derived from diploid *Triticum* and *Aegilops* genomes. The nucleotide sequence distances were computed using the Maximum Composite Likelihood method [53] and are in the units of the number of base substitutions per site. All positions containing gaps and missing data were eliminated from the dataset (Complete deletion option). There were in total 193 positions in the final dataset. The tree optimal (sum of branch length = 0.739407630) is drawn to scale, with branch lengths in the same units as those of the evolutionary distances used to infer the phylogenetic tree.

### CD epitope screening

The deduced amino acid sequences of 26 γ-gliadin contigs, encompassing the CD epitope containing regions (domain II and V), representing a total of 717 transcripts, were explored for epitopes and surrounding sequence regions using a text explorer program (PatternResearch, in-house developed tool for extracting sequences with specific sequence motifs from large datasets; I Padioleau, EMJ Salentijn and MJM Smulders, unpublished) after which the output file was analysed in Excel for further calculations and sorting. In addition, the regions of domain II and domain IV that harbor CD epitopes were aligned (amino acid sequences, MEGA4) to detect sequence variation and to reconstruct the genomic organization of γ-gliadins expressed from the respective loci, *Gli-A1*, *Gli-B1* and *Gli-D1*. Sites for tryptic and peptic enzymes were identified with PeptideCutter (at www.expasy.org/tools/peptidecutter).

### T-cell proliferation assay

A DQ2-γ-I epitope specific T clone was generated from small intestinal biopsies of a celiac disease patient (DQ2.5) as described before [15,16,22]. The patient signed an informed consent form which was approved by the hospital ethics committee. Proliferation assays were performed in triplicate in 150 μl Iscove’s Modified Dulbecco’s Medium (Bio Whittaker, Verviers, Belgium) with 10% pooled normal human serum in 96 well flat-bottom plates using 2x104 gluten specific T-cells stimulated with 105 irradiated HLA-DQ2-matched allogeneic peripheral blood mononuclear cells (3000 rad) in the presence or absence of antigen (1–10 μg/ml, TG2 treated 17 mer pepides). After 2 days 3 H-thymidine (0.5 μCi/well) was added to the cultures, and 18–20 hours thereafter the cells were harvested. 3 H-thymidine incorporation in the T-cell DNA was determined with a liquid scintillation counter (1205 Betaplate Liquid Scintillation Counter, LKB Instruments, Gaithersburg, MD).

## Results

### γ-gliadins from the A^b^, S and D genome

**Figure 1:**
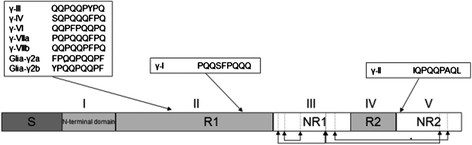
**Schematic structure of a γ-gliadin protein.** Structure of a γ-gliadin protein according to Anderson et al.[36] with the location of the CD epitopes. The protein consists of a short N-terminal signal peptide (S) followed by a unique N-terminal domain (I), two repetitive domains, R1 and R2 (II and IV) and two non-repetitive domains, NR1 and NR2 (III and V). In the first non-repetitive domain and in the second non-repetitive domain respectively six and two conserved cystein residues are present (indicated with vertical lines) that form four interchain disulfide bonds (indicated with arrows). The minimal T-cell epitopes are shown and their approximate position is indicated.

To analyze the genetic diversity of the γ-gliadin gene family a total of 69 different γ-gliadin sequences were cloned from diploid *Triticum* and *Aegilops* species containing the A^b^, S or D genome (Table 1). These diploid genomes were studied because they are related to the ancestors of respectively the A, B and D genome of hexaploid bread wheat (*T. aestivum*). γ-Gliadins contain an N-terminal signal peptide to direct them into the lumen of the endoplasmic reticulum. The mature protein consists of a unique N-terminal domain (Figure 1, domain I) followed by an alternation of two variable domains (Figure 1, domain II and IV) and two more conserved sequence domains (Figure 1, domain III and V). In the conserved domains eight conserved cysteine residues are present that are involved in the formation of intra-molecular disulphide bounds, six in domain III and two in domain V [54]. The six conserved cysteine residues of domain III were present in all sequences whereas no conclusions could be drawn on the cysteine’s in domain V, because this domain was only partly sequenced. Most γ-gliadins from *Ae. speltoides* harboured a ninth conserved cysteine residue on position 14 of domain II [40]. Overall, 6.9% of the clones contained an internal stop codon. The highest percentage of internal stop codons occurred in sequences from *T. monococcum,* in which a quarter (6 out of 16) of all genomic γ-gliadin sequences had a stop codon in domain II of the γ-gliadin sequence due to C to T transitions, generating an in frame truncation after the sequence motif QPQ(Q/L)QFPQPQQP^stop^. 

The genetic variation among the genomic clones was studied in more detail using the Neighbor-Joining method, which resulted in four significantly different groups of genomic γ-gliadin sequences. Two groups contained only sequences derived from *Ae. speltoides*, another group consisted mainly of sequences derived from *Ae. tauschii* together with a few sequences (JQ269782-JQ269785) from *Ae. speltoides,* and the fourth group contained the *T. monococcum* sequences. The number of base substitutions per site among genomic γ-gliadin sequences depended on the species analyzed. For example, all sequences from *Ae. tauschii* showed an average number of base substitutions of 0.216 per site (21.6 base alterations per 100 base pairs) whereas the level of genetic diversity of γ-gliadin sequences derived from *Ae. speltoides* and *T. monococcum* was higher with respectively 0.423 and 0.789 base substitutions per site.

### γ-gliadin transcripts assigned to the A, B and D genome of bread wheat

To analyze the genetic diversity of actively expressed γ-gliadins in bread wheat (*T. aestivum*), γ-gliadin transcripts (939; cDNAs and expressed sequence tags) were extracted from the NCBI database. Of the γ-gliadin transcripts thus obtained 717 were found to code for 26 different γ-gliadin protein isoforms, each represented by contigs with at least four transcripts at a homology level of at least 98%. The number of transcripts for each isoform ranged from 4 to 220, indicating large differences in relative expression rates and redundancy as a part of the differences can be related to different alleles coding for the same amino acid sequence (protein isoform).

**Figure 2:**
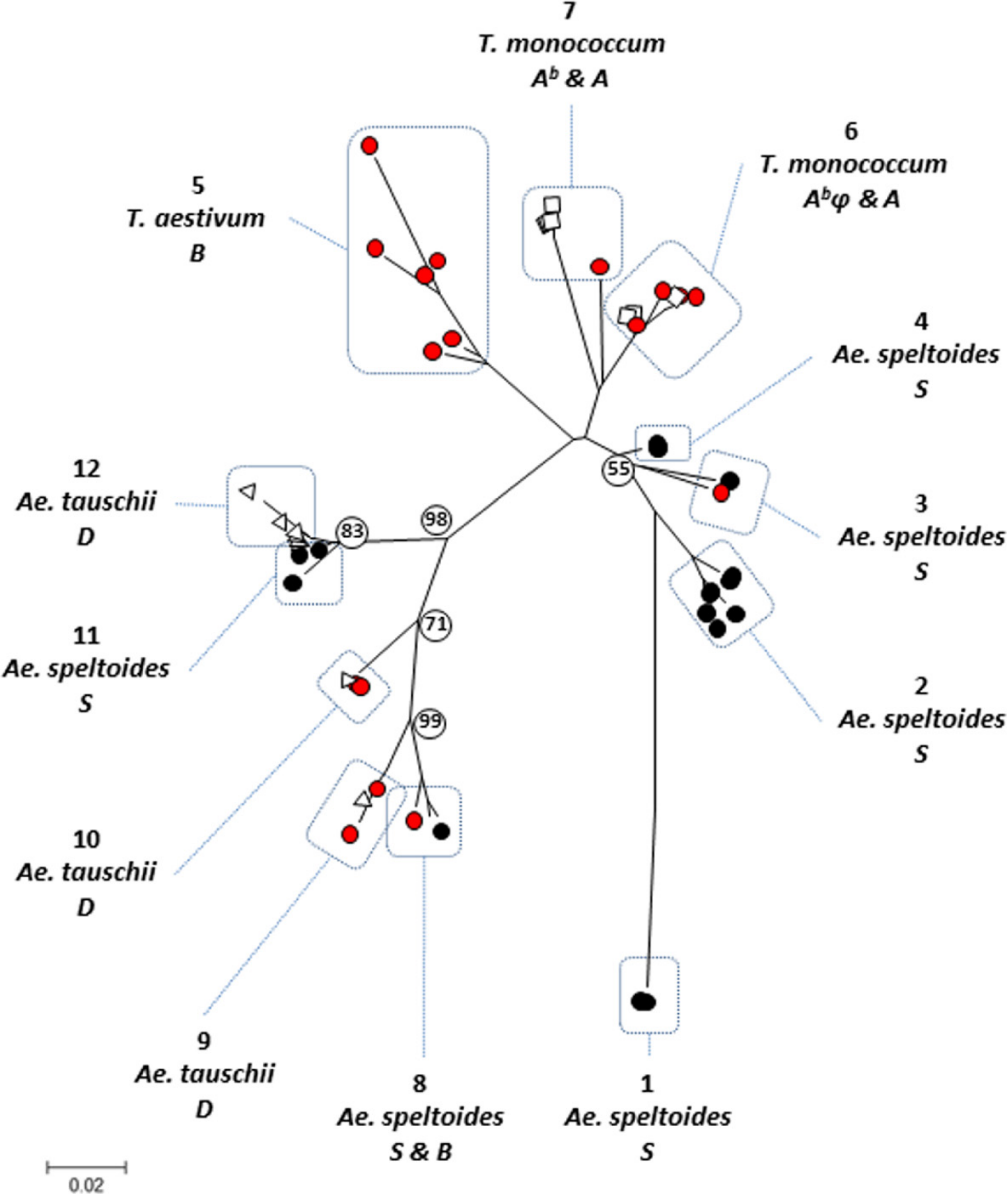
**Neighbor-Joining relationships of γ-gliadin sequences.** Neighbor-Joining relationship of γ-gliadin nucleotide sequences including transcript-contigs of bread wheat (*T. aestivum*, ABD genome, red circles) and genomic γ-gliadin sequences derived from diploid wheat species; *T. monococcum* (A^b^, squares), *Ae. speltoides* (S, black circles), *Ae. tauschii* (D, triangles). In all cases, a region of the γ-gliadin gene, shared by all transcripts sequences and genomic sequences, encompassing domain III, IV and V was analyzed. Numbers on branch points indicate the percentage of replicate trees in which the associated taxa clustered together in the bootstrap test (500 replicates).

Subsequently, the 26 γ-gliadin isoforms of modern bread wheat were assigned to a genomic locus (*Gli-A1*, *Gli-B1* or *Gli-D1*). To realize this, the consensus nucleotide sequences were studied for sequence distances by Neighbor-Joining analysis (domain III, IV and V; 333 to 384 base pairs) together with the ancestrally related genomic γ-gliadin sequences from diploid *Triticum* and *Aegilops* species obtained in these studies, which are carrying the A^b^, S or D genome (related to respectively the A, B and D genome from *T. aestivum*). In the resulting Neighbor-Joining tree 12 topology groups of γ-gliadins could be distinguished (Figure 2). The very center of the tree has low bootstrap values, due to the fact that several branches are joined here at almost the same point, but in varying topology. The different γ-gliadin transcript contigs of bread wheat showed up in seven topology groups (Figure 2, group 3, 5, 6, 7, 8, 9 and 10, red dots). Taking into account the number of transcripts represented by a consensus sequence, almost half (49%, 354 ESTs out of 717 ESTs) of the γ-gliadin transcriptome of bread wheat clustered with D-genomic sequences (Figure 2, group 9 and 10) of *Ae. tauschii* and they were therefore assigned to the γ-gliadin locus *Gli-D1*. The other transcripts were assigned evenly to the other homoeologous loci: 25% to locus *Gli-A1* on basis of their clustering with A^b^-genome sequences derived from *T. monococcum* (Figure 2, group 6 and 7), and 25% to locus *Gli-B1* either by grouping with genomic sequences of *Ae. speltoides* (S genome, 8%, group 3 and 8) or by BlastN homology to *Ae. searsii* (S^s^ genome, 17%, group 5).

### CD epitopes of T.aestivum γ-gliadins in their natural context

In γ-gliadins several CD-immunogenic peptides have been identified (Table 2) and an analysis on the basis of sequence identity showed that all groups of γ-gliadin transcripts of bread wheat contained CD epitope cores but with a different frequency (Table 3). With a frequency of 10.1 CD epitope-cores per transcript, the *Gli-D1* transcripts were coding for the highest number of γ-gliadin CD epitopes followed by the *Gli-A1* transcripts (8.6) and the *Gli-B1* transcripts (5.4). As the *Gli-D1* transcripts were also the highest expressed ones, most CD epitopes found in the γ-gliadin transcriptome originated from *Gli-D1* transcripts (in total 3588 out of 6006 epitope cores or 59%) followed by *Gli-A1* (1533 epitope cores or 25%) and *Gli-B1* (1005 epitope cores or 16%) (Table 3).

**Table 2:** CD-epitope cores derived from γ-gliadins

**epitope**	new epitope nomenclature**	**9-mer core**
DQ2-γ-VIIb^3*^	DQ2.5-glia-γ4c	**Q**QP**Q**QPFPQ
DQ2-γ-VI^3^	DQ2.5-glia-γ5	**Q**QPFP**Q**QPQ
DQ2.5-glia-γ2a^6^	x	FP**Q**QP**Q**QPF
DQ2-γ-II(Glia γ30)^3,4^	DQ2.5-glia-γ2	IQP**Q**QPAQL
DQ2-γ-I (Glia-γ1)^2^	DQ2.5-glia-γ1	P**Q**QSFPQQ**Q**
DQ2-γ-VIIa^3^	DQ2.5-glia-γ4b	PQP**Q**Q**Q**FPQ
DQ2-γ-IV^5^	DQ2.5-glia-γ4a	SQP**Q**Q**Q**FPQ
DQ2-γ-III^5*^	DQ2.5-glia-γ3	**Q**QP**Q**QPYP**Q**
DQ2-glia-γ2b^6^	x	YP**Q**QP**Q**QPF

CD-epitope cores (9-mer binding cores) derived from γ-gliadins. The targets for tTG deamidation are depicted in **bold underlined**. ^2^Sjöström et al. [17], ^3^Qiao et al.[7], ^4^Vader et al.[22], ^5^Arentz-Hansen et al [19], ^6^Stepniak et al.[21]. *deamidation dependent epitope, **Q**QP**Q**QPF/YP**Q**, deamidation at position 4 is HLA-DQ2 restricted and deamidation at positions 1 and 9, **Q**QP**Q**QP(F/Y)P**Q** is HLA-DQ8 [8]; the same counts for DQ2-γ-VI (**Q**QPFP**Q**QP**Q**), in case of deamidation at P1 and P9 this epitope is HLA-DQ8 restricted [9]. ** Nomenclature and listing of celiac disease relevant gluten T-cell epitopes (Sollid et al. [55]).

**Table 3:** **The number of CD-epitope cores in γ-gliadin transcripts of*T. aestivum***

**epitope**	***Gli-A1***	***Gli-A1***	***Gli-B1***	***Gli-B1***	***Gli-B1***	***Gli-D1***	***Gli-D1***	**∑**
	**Group 6**	**Group 7**	**Group 3**	**Group 5**	**Group 8**	**Group 9**	**Group 10**	
DQ2-γ-VIIb^3*^	701	144	72	230	57	1257	50	2511
DQ2-γ-VI^3^	224	0	0	0	38	678	50	990
DQ2-glia-γ2a^6^	140	30	36	59	38	684	0	987
DQ2-γ-II(Glia γ30)^3,4^	136	0	41	125	0	293	61	656
DQ2-γ-I (Glia-γ1)^2^	120	38	41	115	19	293	61	567
DQ2-γ-VIIa^3^	0	0	0	115	19	0	0	134
DQ2-γ-IV^5^	0	0	0	0	0	0	61	61
DQ2-γ-III^5*^	0	0	0	0	0	0	50	50
DQ2-glia-γ2b^6^	0	0	0	0	0	0	50	50
N_epitopes_	*1321*	*212*	*190*	*644*	*171*	*3205*	*383*	*6006*
N_transcripts_	***140***	***38***	***41***	***125***	***19***	***293***	***61***	*717*
N_epitopes/_N_transcripts_	*9.4*	*5.6*	*4.6*	*5.2*	*9.0*	*10.9*	*6.3*	*8.4*
N_epitopes/_N_transcripts_	*8.6 (1533/178)*		*5.4 (1005/185)*			*10.1 (3588/354)*		

The number of T-cell stimulating sequences involved in CD (9 mer epitope cores) found in a set of 717 γ-gliadin transcripts from *T. aestivum* (genome ABD) attributed to respectively *Gli-A1* (topology group 6 and 7), *Gli-B1* (topology group 3, 5, and 8) and *Gli-D1* (topology group 9 and10). The sequence topology was obtained by Neighbor joining analysis (see Figure 2). The frequency of T-cell epitopes in γ- gliadin transcripts (N_epitopes/_N_transcripts_) is calculated.

**Figure 3:**
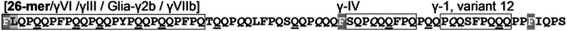
**CD-epitopes of γ-gliadin transcripts of* T. aestivum* in their natural context.** The deduced aminoacid sequences of a *T.aestivum* γ-gliadin transcripts (N = 61) assigned to locus *Gli-D,* Neighbor Joining topology group 10, spanning a part of the first repetitive domain of the γ-gliadin sequence. CD T-cell epitopes are depicted: γ-I (PQQSFPQQQ), γ-III (QQPQQPYPQ), γ-IV (SQPQQQFPQ), γ-VI (QQPFPQQPQ), γ-VIIb (QQPQQPFPQ), 26-mer (FLQPQQPFPQQPQQPYPQQPQQPFPQ). Glutamine residues that are a primary targets for the enzyme tissue transglutaminase are underlined (**Q**) in QxP target sites whereas moderate target sites are depicted in italics (*Q*) [13,14]. Cleavage sites: in grey with white letters, chymotrypsin-high specificity; in grey with black letters, chymotrypsin-low specificity; cleavage occurs at the right side (C-terminal direction) of the marked amino acid.

By analyzing the CD epitopes in their natural context, distinct differences in genomic organization and epitope composition were observed among bread wheat γ-gliadin transcripts derived from the three γ-gliadin loci, *Gli-A1*, *Gli-D1* and *Gli-B1* (Additional file 2, Figure 3). In many cases, repeat motifs contained overlapping CD epitopes. For instance, transcripts expressed from *Gli-D1* contained a repeat motif of 17 amino acids (**Q**QP**Q**QPFP**Q**QP**Q**QPFPQ; underlined Q residues are target sites for the enzyme tissue transglutaminase) that included three overlapping CD epitopes (DQ2-γ-VIIb, DQ2-Glia-γ2a, DQ2-γ-VI). Because predicted tryptic cleavage sites were absent in these repeats they are most likely resistant to enzymatic degradation after ingestion. Indeed, a sequence of 26 amino acids present in a part of the *Gli-D1* transcripts (Figure 3), 9% of the overall γ-gliadin transcriptome and harbouring four CD-epitope cores (FLQP**Q**QPFP**Q**QP**Q**QPYP**Q**QP**Q**QPFPQ) has been reported to be resistant to proteolysis [56]. Such CD epitope-rich repeat motifs were also present in transcripts expressed from *Gli-A1* and *Gli-B1*, despite the shorter repetitive domain of *Gli-B1* transcripts (Additional file 2). The genomic structure of the C-terminal domain V is less complex and harbours a single CD epitope, DQ2-γ-II (glia-γ30) (IQP**Q**QPAQL) (Table 4). Only two variants representing 9% of the transcripts displayed an amino acid change within the 9-mer core (L9 to Y9 and P6 to L6). 

**Table 4:** **The DQ2- γ-II epitope in transcripts of*T.aestivum***

	_**1 2 3 4 5 6 7 8 9**_		**locus**	**group**	**%**
LVQG*Q*GI	IQP**Q**QPA*Q*L	EVI**R**SLVLGTLPTMCN	***Gli-A1***	6	19
LVQG*Q*GI	IQP**Q**QPA*Q*Y	EVI**R**SLVL**R**TLPNMCN	***Gli-A1***	7	5
LVQG*Q*GI	IQP**Q**QPA*Q*L	EVI**R**SLVL**R**TLPTMCN	***Gli-B1***	3	6
LVQG*Q*GI	IQP*Q*QLAQL	EAI**R**SLVLQTLPTMCN	***Gli-B1***	8	3
LAQGLGI	IQP**Q**QPAQL	EGI**R**SLVL**K**TLPTMCN	***Gli-B1***	5	17
LVQG*Q*GI	IQP**Q**QPAQL	EAI**R**SLVLQTLPSMCN	***Gli-D1***	9	41
LVQG*Q*GI	IQP**Q**QPAQL	EAI**R**SLVLQTLPTMCN	***Gli-D1***	10	9

Natural variants of γ-II (glia-γ30; IQP**Q**QPAQ) epitope in expressed γ-gliadins of *T.aestivum*. This epitope is located in the C-terminal part of γ-gliadins (domain V). % = fraction of total number of γ-gliadins transcripts. Glutamine residues that are a primary targets for the enzyme tissue transglutaminase are to depicted in bold (**Q**) in QxP target sites whereas moderate target sites are depicted in italics, (*Q*) [13,14]. Cleavage sites: **R, K** in bold, trypsin; cleavage occurs at the right side (C-terminal direction) of the marked amino acid.

### Immunogenicity of DQ2-γ-I epitope variants

The DQ2-γ-I epitope (P***Q***QSFPQQ**Q**) found in the variable domain II of γ-gliadins is known as a major CD epitope derived from the γ-gliadin fraction of gluten [17,22,24]. In the present study thirteen natural variants of the DQ2-γ-I epitope (including the 9-mer core motif and four flanking amino acid positions on both sides of the epitope core) were found among genomic sequences of *T. monococcum* subspec. monococcum (A^b^ genome), *Ae. speltoides* var. speltoides (S-genome), *Ae. tauschii* (D-genome), and *T. aestivum* transcripts (ABD genome) (Table 5). According to the rules for enzymatic TG2 deamidation [13,14], position Q2 of the DQ2-γ-I epitope core (P***Q***QSFPQQ**Q**) is a moderate target for TG2 whereas the genetic variation at the C-terminal flanking positions +1 to +3 can influence the deamidation pattern of Q7 to Q9. Based on the natural variation in this flanking region several different deamidation profiles of the DQ2-γ-I epitope core are evident (Table 5 and 6). These variants were deamidated by TG2 and tested for their capacity to stimulate a DQ2-γ-I epitope specific T-cell clone (Table 6). The results show that variants in which Q2 and Q9 are deamidated stimulate the T-cell clone while variants in which Q8 instead of Q9 is deamidated or the F5 is replaced by a S do not display such activity. Thus, natural variants of the DQ2-γ-I epitope exist that influence the T-cell stimulatory capacity. 

**Table 5:** Natural variants of the DQ2-γ-I epitope

**No.**	**4**^**-**^	**3**^**-**^	**2**^**-**^	**1**^**-**^	**1**	**2**	**3**	**4**	**5**	**6**	**7**	**8**	**9**	**1**^**+**^	**2**^**+**^	**3**^**+**^	**4**^**+**^	**Genome**	**Group**
1	Q	P	**Q**	Q	P	*Q*	Q	S	F	P	Q	Q	**Q**	Q	P	L	I	ABD	3, 7
2	Q	P	**Q**	Q	P	*Q*	Q	S	F	P	Q	*Q*	*Q*	*Q*	L	M	I	ABD	5
3*	Q	P	**Q**	Q	P	**Q**	Q	P	F	P	Q	Q	**Q**	Q	P	L	I	ABD, A^b^	6
4	Q	P	**Q**	Q	P	*Q*	Q	S	F	P	Q	Q	**Q**	Q	P	A	I	ABD	5
5	Q	P	**Q**	Q	P	*Q*	Q	S	F	P	Q	**Q**	Q	P	S	L	I	D, S	12, 11
6	Q	P	**Q**	Q	P	Q	Q	S	S	P	Q	*Q*	*Q*	*Q*	L	L	I	S	1, 2
7	Q	S	**Q**	Q	P	Q	Q	S	S	P	Q	*Q*	*Q*	*Q*	L	L	I	ABD, S	3
8	Q	P	Q	Q	S	Q	Q	S	S	P	Q	*Q*	*Q*	*Q*	L	L	I	S	4
9	Q	P	**Q**	Q	P	*Q*	Q	S	F	P	Q	*Q*	*Q*	*Q*	W	M	I	ABD, S	5
10	Q	P	**Q**	Q	P	*Q*	Q	S	F	P	Q	Q	**Q**	R	P	F	I	ABD, S, D	8, 9
11	Q	P	**Q**	Q	P	*Q*	Q	S	F	P	Q	Q	*Q*	**R**	S	F	I	ABD, D	9
12	Q	P	**Q**	Q	P	*Q*	Q	S	F	P	Q	**Q**	Q	P	P	F	I	ABD, D	10
13	Q	P	**Q**	Q	P	*Q*	Q	S	F	P	Q	**Q**	Q	P	P	L	I	A^b^	7

Natural variants of the DQ2-γ-I epitope, including the 9-mer core motif and four flanking amino acid positions on both sides of the core, found among genomic sequences of *T. monococcum* subspec. monococcum (A^b^ genome), *Ae. speltoides* var. speltoides (S-genome) and *Ae. tauschii* (D-genome) and *T. aestivum* transcripts (ABD genome). Group = Neighbor-Joining topology group of the epitope motif (see Figure 2). Glutamine residues that are a primary targets for the enzyme tissue transglutaminase are bold underlined (**Q**) in QxP target sites whereas moderate target sites are depicted in italics, underlined (*Q*) [13,14]. Cleavage sites: **R** in bold, trypsin; F underlined, chymotrypsin-high specificity; cleavage occurs at the right side (C-terminal direction) of the marked amino acid.

**Table 6:** T-cell stimulating capacity of DQ2-γ-I is influenced by deamidation pattern and flanking amino acids

**1**	**2**	**3**	**4**	**5**	**6**	**7**	**8**	**9**	**SI**	**Peptide no.**
P	*Q*	Q	S	F	P	Q	Q	**Q**	+	1,4
P	**Q**	Q	P	F	P	Q	Q	**Q**	+	3
P	*Q*	Q	S	F	P	Q	*Q*	*Q*	++	2
P	*Q*	Q	S	F	P	Q	**Q**	Q	-	5,12,13
P	Q	Q	S	S	P	Q	*Q*	*Q*	-	6,7
S	Q	Q	S	S	P	Q	*Q*	*Q*	-	8

Stimulation of a T-cell clone (SIM 114, DQ2.5) specific for CD epitope DQ2-γ-I, with deamidated 17 mer peptides (see Table 5) that are representing natural occurring variants of DQ2-γ-I found in γ-gliadin transcripts of bread wheat (ABD, *T. aestivum*) and ancestrally related diploid wheat genomes (A^b^, S and D). Peptide no. corresponds to the peptide numbering in Table 5. Glutamine residues that are a primary targets for the enzyme tissue transglutaminase are underlined (**Q**) in QxP target sites whereas moderate target sites are depicted in italics (*Q*) [13,14]. The stimulation is mediated by HLA-DQ2 carrying antigen presenting cells (APC). SI = Stimulation index = counts per minute (cpm) of the stimulated culture/cpm unstimulated culture (with APC only); ++ = SI ≥ 50, + = 20 ≤ SI <50, ± =10 ≤ SI <20, - = SI <10.

## Discussion

Wheat gluten is the source of a large repertoire of T-cell epitopes involved in the pathogenesis of CD. It is clear that most patients react to epitopes derived from α-gliadins. However, there is increasing evidence that more T-cell epitopes from other gluten genes are involved in CD [24,57]. It was shown that next to α-gliadins also T-cell epitopes from γ-gliadins play a significant role in the development of CD [2,17-19,22,24]. Tye-Din et al. [57] found that gluten specific polyclonal T-cells in the peripheral blood of CD patients were specific for the same set of gluten peptides after feeding the patients with the same cereal but after the patients ingested respectively wheat, barley and rye different T-cells, recognizing different sets of gluten peptides, were found. Also, it was hypothesized that a T-cell response to gluten can be initiated against a relatively large number of peptides, and when the response evolves it focuses on the most immuno-dominant sequences [22]. So, there are some indications that the composition of different cereals and wheat varieties in the diet [24,57] as well as the disease status [22] may play a role in the defining the pattern of T cell reactivity towards different gluten peptides in a celiac patient.

Using polyclonal, gliadin reactive T-cell lines, Camarca et al. [24] showed that T-cell reactivity towards the γ-gliadin derived peptides is more heterogeneous compared to the reaction to α-gliadin derived peptides; reaction to the latter was focused towards a few immunodominant peptides whereas a larger repertoire of different γ-gliadin derived peptides was found and patients were recognizing varying sets of peptides. They suggested that this less focussed behaviour of γ-gliadin peptides in celiac disease may reflect the high genetic diversity of γ-gliadins.

To obtain insight in CD epitopes derived from γ-gliadins we performed a detailed study of the presence and natural variation of CD epitopes derived from the γ-gliadin gluten fraction of bread wheat (*T. aestivum L.*) which is an allo-hexaploid (2n = 6x = 42) carrying three homoeologous genomes A, B and D. This study included two datasets: (1) 69 novel genomic γ-gliadin clones from the diploid wheat species *T. monococcum*, *Ae. speltoides* and *Ae. tauschii.* These diploid wheat species are carrying respectively genome A^b^, S and D which are ancestrally related to the three respective homoeologous genomes of bread wheat, A, B and D; and (2) γ-gliadin transcripts from bread wheat (*T. aestivum*) derived from the public database (Genbank, NCBI)*.* By similarity analysis the transcripts of bread wheat could be assigned to locus *Gli-A1, Gli-B1*, or *Gli-D1* (on the respective homoeologous chromosomes 1A, 1B and 1D). To reduce the complexity of the multigene γ-gliadin family, transcripts of bread wheat were analyzed (instead of genomic sequences). As such, the γ-gliadin transcriptome is a minimal estimate for the γ-gliadin variants in gluten of bread wheat. The γ-gliadin transcripts of bread wheat (N = 717) encode for 26 different γ-gliadin protein isoforms of which ten represented full length γ-gliadins (contigs >300 bp, ≥ four transcripts, 98% to 99% sequence identity). This figure is in keeping with the 15–40 estimated γ-gliadin genes in hexaploid wheat estimated before [35,36,41]. Half of the γ-gliadin transcripts from bread wheat grouped with *Ae. tauschii* sequences (D genome, 6 contigs, 354 transcripts) and were assigned to the γ-gliadin locus on chromosome 1D (*Gli-D1*), indicating that the majority of the γ-gliadins are expressed from the D genome. The remainder of the transcripts were equally assigned to *Gli-A1* (9 contigs, 178 transcripts) and *Gli-B1* (11 contigs, 185 transcripts). Similarly, for the α-gliadins it was found that the highest expression occurred from the D genome (*Gli-D2*) [58].

With regard to CD epitope content, our analysis shows that γ-gliadins from bread wheat contain on average between 4 and 10 potential CD epitopes in the first variable domain (Table 3, range 4.6 to10.9 epitope cores per transcript). The 26-mer γ-gliadin peptide that harbours four distinct CD epitopes [56] is only present in D-genome γ-gliadins. It is present in 9% of the γ-gliadin transcripts of bread wheat. Thus, the γ-gliadins from all three genomes encode a large number of different immunogenic peptides which exceeds the number of identified immunogenic peptides in the α-gliadins. Perhaps this is the basis of the observation that immuno-dominant responses to α-gliadin derived CD epitopes are generally found in patients, while responses to the γ-gliadins appear less consistent as was previously suggested by Camarca et al. [24]. If patients make frequently T-cell responses to a limited number α-gliadin peptides but can respond to a large number of different γ-gliadin derived peptides, T-cells for some specific α-gliadin epitopes may prevail numerically but collectively γ-gliadin derived T-cell epitopes are probably similarly effective in causing CD.

The DQ2-γ-I epitope (P*Q*QSFPQQ**Q**) is regarded as a major CD epitope as it is frequently recognized by CD patients [22,24]. We observed a high level of natural genetic variation in the C-terminal flanking sequence of this CD epitope. According to the rules for deamidation by TG2 [13,14] the variation in the C-terminal flanking sequences determines the deamidation pattern of this epitope core. Among diploid and hexaploid wheat six variants of the 9-mer DQ2-γ-I epitope core were evident with respect to variation in amino acid composition and deamidation pattern. Testing of these DQ2-γ-I variants for their capacity to trigger proliferation of DQ2-γ-I specific T-cells confirmed that TG2 deamidation of glutamine (Q) at position 9 is essential for T-cell stimulation, most likely because it is providing a negative charge which is essential for HLA-DQ2 binding. Variant peptides that displayed a serine (S) at position 5 instead of an phenylalaninine (F) were found to have lost T-cell stimulatory capacity. Moreover, the presence of a positively charged arginine (R) residue at position +1 diminished T-cell proliferation (Additional file 3), most likely because it influenced the deamidation of Q9 as previously observed by Dørum et al. [28]. Similarly, a tryptophan (W) at position +2 inhibited the T-cell response (Additional file 3). Thus, both substitutions within and outside the epitope core can influence the T-cell stimulatory properties of the DQ2-γ-I epitope.

Taken together, the γ-gliadins from all three wheat genomes appear to be a significant source of CD epitopes. Similar to the α-gliadins, the highest number of potential immunogenic γ-gliadin peptides are encoded by the D genome of bread wheat which is thus the most critical genome with regard to CD toxicity. However, gluten derived from tetraploid wheat varieties lacking the D genome is not tolerated by CD patients as well, indicating that the mere elimination of the D genome is not sufficient for the generation of safe wheat. Regarding the A-genome of wheat, there are indications for the existence of *T. monococcum* spp. with a level or type of gliadin that is unable to induce IFN-γ production and histologic damage as was observed for instance in duodenal biopsy specimens from patients with celiac disease [59]. So, the high level of genetic variation among wheat lines and the presence of genetic variation influencing the immunogenicity of the major CD epitopes may offer possibilities to generate wheat varieties with a reduced CD-immunogenicity, tailored to major CD epitopes (i.e. DQ2-α-I, DQ2-α-II; [46] and/or DQ2-γ-I, this study). Such varieties would help to reduce the presence of immunogenic CD epitopes in wheat flour and, while not safe for consumption by patients, might help to prevent the onset of CD in people that carry genetic risk factors [44,45]. However, introgression from the diploid to the hexaploid level is a time-consuming process, and consequently it will take many years before the product of such a synthetic hexaploidisation has been bred to sufficient agronomic quality. Alternatively, one could screen a large number of existing varieties for differences in their toxicity for CD patients as the analysis of CD epitope regions in transcript sequences does provide an accurate and quick method for screening [46,58]. Consequently, we are currently analyzing CD epitope regions in the gluten transcriptome of tetraploid wheat cultivars in a medium-throughput way by employing next-generation sequencing technology.

## Conclusions

The results presented here for γ-gliadins form a significant contribution to the approach whereby the CD toxicity of wheat cultivars is quantified directly from protein-coding mRNA sequences.

## Competing interest

The authors declare that they have no competing interest.

## Authors’ contributions

EMJS and MJMS designed the studies. EMJS carried out sequence analysis and wrote the manuscript. DCM carried out T-cell tests. SVG performed cloning and sequence analysis. ISMP and carried out sequence analysis and bioinformatics. FK and MJMS helped to coordinate the study and draft the manuscript. LJWJG and IMM helped to draft the manuscript. All authors read and approved the final manuscript. ISMP is currently employed at Université de Genève as a bio-informatician. All authors read and approved the final manuscript.

## Supplementary Material

Additional file 1 **Ten full-length γ-gliadin sequences.** Ten full length γ-gliadin nucleotide sequences obtained after the assemblage of 717 γ-gliadins transcripts at 98% homology. In brackets, the number of sequences in a contig. Click here for file

Additional file 2 **CD-epitopes of γ-gliadin transcripts of* T. aestivum* in their natural context.** Alignments of the deduced aminoacid sequences of *T.aestivum* γ-gliadin transcript contigs (717 transcripts from the Genbank NCBI) spanning a part of the first repetitive domain and a part of the γ-gliadin sequence. CD T-cell epitopes are depicted in bold: γ-I (PQQSFPQQQ), γ-III (QQPQQPYPQ), γ-IV (SQPQQQFPQ), γ-VI (QQPFPQQPQ), γ-VIIa (PQPQQQFPQ), γ-VIIb (QQPQQPFPQ), Glia-γ2a (FPQQPQQPF), 26-mer FLQPQQPFPQQPQQPYPQQPQQPFPQ. *Gli-A*: *T.aestivum* γ-gliadin transcripts expressed from locus *Gli-A1*, Neighbor Joining topology group 6 (N = 140 transcripts) and 7 (N = 38 transcripts). *Gli-B*: *T.aestivum* γ-gliadin transcripts expressed from locus *Gli-B1*, Neighbor Joining topology group 3 (N = 41 transcripts), 5 (N = 125 transcripts) and 8 (N = 19 transcripts). *Gli-D*: *T.aestivum* γ-gliadin transcripts expressed from locus *Gli-D1*, Neighbor Joining topology group 9 (N = 293 transcripts) and 10 (N = 61 transcripts). Alignment gaps are indicated with dashes (−). Shorter sequences, not connected to domain I are marked with #. Glutamine residues that are a primary targets for the enzyme tissue transglutaminase are underlined (Q) in QxP target sites whereas moderate target sites are depicted in italics (*Q*) [13,14]. Variants of DQ2-γ-I are indicated. Cleavage sites: In black, trypsin; in grey with white letters, chymotrypsin-high specificity; in grey with black letters, chymotrypsin-low specificity; cleavage occurs at the right side (C-terminal direction) of the marked amino acid. Click here for file

Additional file 3 *** In vitro* T-cell stimulating capacity of natural variants of celiac disease epitope, DQ2-γ-I.** Stimulation of a T-cell clone specific for CD epitope DQ2-γ-I, with natural occurring variants of DQ2-γ-I (9-mer epitope core PQQSFPQQQ and residues in the positions −1 to −4 and +1 to +4). Glutamine residues that are a primary targets for the enzyme tissue transglutaminase are underlined (Q) in QxP target sites whereas moderate target sites are depicted in italics (*Q*) [13,14]. SI = Stimulation Index = cpm of the stimulated culture/cpm unstimulated culture (with APC only); ++ = SI ≥ 50, + = 20 ≤ SI <50, ± =10 ≤ SI <20, - = SI <10. The stimulation is mediated by HLA-DQ2 carrying antigen presenting cells (APC). Peptide no. corresponds to the peptide numbering in Table 5. Click here for file
