# Supplementary material for: Celiac disease T-cell epitopes from gamma-gliadins: immunoreactivity depends on the genome of origin, transcript frequency, and flanking protein variation
Source: BMC Genomics. 2012 Jun 22;13:277. doi: 10.1186/1471-2164-13-277 (PMC3469346; doi:10.1186/1471-2164-13-277)
Supplement: Additional file 2 — CD-epitopes of γ-gliadin transcripts of T. aestivum in their natural context. Alignments of the deduced aminoacid sequences of T.aestivum γ-gliadin transcript contigs (717 transcripts from the Genbank NCBI) spanning a part of the first repetitive domain and a part of the γ-gliadin sequence. CD T-cell epitopes are depicted in bold: γ-I (PQQSFPQQQ), γ-III (QQPQQPYPQ), γ-IV (SQPQQQFPQ), γ-VI (QQPFPQQPQ), γ-VIIa (PQPQQQFPQ), γ-VIIb (QQPQQPFPQ), Glia-γ2a (FPQQPQQPF), 26-mer FLQPQQPFPQQPQQPYPQQPQQPFPQ. Gli-A: T.aestivum γ-gliadin transcripts expressed from locus Gli-A1, Neighbor Joining topology group 6 (N = 140 transcripts) and 7 (N = 38 transcripts). Gli-B: T.aestivum γ-gliadin transcripts expressed from locus Gli-B1, Neighbor Joining topology group 3 (N = 41 transcripts), 5 (N = 125 transcripts) and 8 (N = 19 transcripts). Gli-D: T.aestivum γ-gliadin transcripts expressed from locus Gli-D1, Neighbor Joining topology group 9 (N = 293 transcripts) and 10 (N = 61 transcripts). Alignment gaps are indicated with dashes (−). Shorter sequences, not connected to domain I are marked with #. Glutamine residues that are a primary targets for the enzyme tissue transglutaminase are underlined (Q) in QxP target sites whereas moderate target sites are depicted in italics (Q) [13,14]. Variants of DQ2-γ-I are indicated. Cleavage sites: In black, trypsin; in grey with white letters, chymotrypsin-high specificity; in grey with black letters, chymotrypsin-low specificity; cleavage occurs at the right side (C-terminal direction) of the marked amino acid. [file 1471-2164-13-277-S2.pdf]

Gli-A1	Group	%
γVIIb/γ-VI · γVIIb/γ-VI · γVIIb · Glia-γ2a/γVIIb · γVIIb · γVIIb/γ-I (variant 3)<br>QPQQQFPQPQQPQQPFPQQPQQQFPQPQQPQQPFPQQPQQQFPQPQQPQQPFPQPQQPQLPFPQQPQQPFPQPQQPQQPFPQLQQPQQPLPQPQQPQQPFPQQQQPLIQPY	6	16
Glia-γ2a/γVIIb · γVIIb · γVIIb/γ-I (variant 3)<br>Q---PQQPFPQPQQPQLPFPQQPQQPFPQPQQPQQPFPQLQQPQQPLPQPQQPQQPFPQQQQPLIQPY	6	1
γVIIb/γ-VI · γVIIb · Glia-γ2a/γVIIb<br>#PQQQFPQPQQPQ---------QQFPQPQQPQQPFPQQPQQQFPRPQQPQQPFPQPQQPQLPFPQQPQQPFPQPQQPQQPFSQLQQPQQPLPQPQQSQQPFSQQQQSLIQPY	6	3
γVIIb · Glia-γ2a/γVIIb · γVIIb · γVIIb · γ-I (variant 1)<br>#PQQQFPQPQQPQ---------------------------QQFPQPQQPQQPFPQPQQAQLPFPQQPQQPFPQPQQPQQPFPQSQQPQQPFPQPQQPQQSFPQQQQPLIQPY	7	4
γVIIb · γVIIb · γVIIb · γ-I (variant 1)<br>G---QQPQQPFPQPQQPQQPFPQSQQPQQPFPQPQQPQQSFPQQQQPLIQPY	7	1
Gli-B1	Group	%
γ-VI/Glia-γ2a · γVIIb/γ-VI/Glia-γ2a · γVIIb · γ-VIIa · γ-I (variant 10)<br>LQPQQPFPQQPQQPFPQTQQPQQPFPQQPQQPFPQTQQPQQPFPQFQQPHQPFPQPQQQFPQPQQPQQSFPQQQRPFIQPS	8	3
Glia-γ2a /γVIIb · γVIIb · γ-I (variant 1)<br>#FPQQP---------------QLPFPQQPQQPFPQPQQPQQQFPQSQQPQQPFPQPQQQFLQPQQPQQSFPQQQQPLIQLS	3	6
Glia-γ2a /γVIIb · γVIIb · γVIIb/γ-VIIa · γ-I (variant 9)<br>#QPQQPFPQPQQP--------QLPFPQQPQQPFPQPQQPQQPFPQSQQPQQPFPQPQQQFPQPQQPQQSFPQQQQWMIQSF	5	1
Glia-γ2a /γVIIb · γVIIb · γVIIb/γ-VIIa · γ-I (variant 4)<br>#-QQP----------------QLPFPQQPQQPFPQPQQPQQPFPQSQQPQQPFPQPQQQFPQPQQPQQSFPQQQQPAIQSF	5	6
γVIIb · γVIIb/γ-VIIa · γ-I (variant 2)<br>Q------------------------------PFPQPQQPQQPFPQSQQPQQPFPQPQQQFPQPQQPQQSFPQQQQLMIQSF	5	4
γVIIb · γVIIb/γ-VIIa · γ-I (variant 4)<br>Q----------------------------------PQQPQQPFPQSQQPQQPFPQPQQQFPQPQQPQQSFPQQQQPAIQSF	5	5
Gli-D1	Group	%
Glia-γ2a/ γVIIb · γVIIb/γ-VI/Glia-γ2a · γVIIb/γ-VI/Glia-γ2a · γVIIb/γ-VI/Glia-γ2a · γVIIb · γVIIb · γ-I, variant 11<br>FLQPQQAFPQQPQQPFPQTQQPQQPFPQQPQQPFPQTQQPQQPFPQQPQQPFPQTQQPQQPFPQQPQQPFPQTQQPQQPFPQLQQPQQPFPQPQQQLPQPQQPQQSFPQQQRSFIQPS	9	1
γVIIb/γ-VI/Glia-γ2a · γVIIb/γ-VI/Glia-γ2a · γVIIb/γ-VI/Glia-γ2a · γVIIb · γVIIb · γ-I, variant 11<br>FLQ------------------PQQPFPQQPQQPFPQTQQPQQPFPQQPQQPFPQTQQPQQPFPQQPQQPFPQTQQPQQPFPQLQQPQQPFPQPQQQLPQPQQPQQSFPQQQRSFIQPS	9	30
γ-I, variant 10<br>FPQ--PQQQLPQPQQPQQSFPQQQRPFIQPS	9	2
γVIIb · γVIIb · γ-I, variant 10<br>Q--------------------------------------PQQPFPHQPQQPFPQTQQPQQPFPKQPQQPFPQTQQPQQPFPQLQQPQQPFPQPQQQLPQPQQPQQSFPQQQRPFIQPS	9	8
[**26-mer**/γVI /γIII / Glia-γ2b / γVIIb] · γ-IV · γ-1, variant 12<br>--FLQPQQPFPQQPQQPYPQQPQQPFPQTQQPQQLFPQSQQPQQQFSQPQQQFPQPQQPQQSFPQQQPPFIQPS	10	9
