# Supplementary material for: Celiac disease T-cell epitopes from gamma-gliadins: immunoreactivity depends on the genome of origin, transcript frequency, and flanking protein variation
Source: BMC Genomics. 2012 Jun 22;13:277. doi: 10.1186/1471-2164-13-277 (PMC3469346; doi:10.1186/1471-2164-13-277)
Supplement: Additional file 3 — In vitro T-cell stimulating capacity of natural variants of celiac disease epitope, DQ2-γ-I. Stimulation of a T-cell clone specific for CD epitope DQ2-γ-I, with natural occurring variants of DQ2-γ-I (9-mer epitope core PQQSFPQQQ and residues in the positions −1 to −4 and +1 to +4). Glutamine residues that are a primary targets for the enzyme tissue transglutaminase are underlined (Q) in QxP target sites whereas moderate target sites are depicted in italics (Q) [13,14]. SI = Stimulation Index = cpm of the stimulated culture/cpm unstimulated culture (with APC only); ++ = SI ≥ 50, + = 20 ≤ SI <50, ± =10 ≤ SI <20, - = SI <10. The stimulation is mediated by HLA-DQ2 carrying antigen presenting cells (APC). Peptide no. corresponds to the peptide numbering in Table 5. [file 1471-2164-13-277-S3.docx]

**Additional file 3 - *In vitro* T-cell stimulating capacity of natural variants of celiac disease epitope, DQ2-γ-I.**

| _4_^-^_3_^-^_2_^-^_1_^-^ _1 2 3_ **_4_** _5_ **_6_** _7 8 9 1_^+^_2_^+^_3_^+^_4_^+^ | SI |
| --- | --- |
| QP**Q**Q P*Q*QSFPQQ**Q** QPLI | ++ |
| QP**Q**Q P*Q*QSFPQQ**Q** RPFI | - |
|  |  |
| QP**Q**Q P*Q*QSFPQ*QQ* *Q*LMI | ++ |
| QP**Q**Q P*Q*QSFPQ*QQ* *Q*WMI | - |
